# Impact of Cannabis Seed Incorporation in Layer Diet on Productive Performance and Egg Quality Traits

**DOI:** 10.1155/2023/5565825

**Published:** 2023-02-28

**Authors:** Yassine Taaifi, Kamal Belhaj, Farid Mansouri, Youssef Rbah, Najlae Elbouanani, Reda Melhaoui, Abdesamad Ben Moumen, Embarek Azeroual, Hana Serghini-Caid, Ahmed Elamrani

**Affiliations:** ^1^Laboratory for Agricultural Productions Improvement, Biotechnology and Environment, Faculty of Sciences, University Mohammed First, BP-717, 60000 Oujda, Morocco; ^2^Laboratory of Sustainable Agriculture Management, Higher School of Technology Sidi Bennour, University Chouaib Doukkali, Street Jabran Khalil Jabran BP 299-24000, El Jadida, Morocco; ^3^Higher School of Education and Training, Mohammed I University, BP-410, 60000 Oujda, Morocco; ^4^Agroalimentary and Health Laboratory, Faculty of Sciences and Technology, Hassan First University, Hassan, Karnataka, India; ^5^Royal Institute of Livestock Fouarat, Kenitra, Morocco

## Abstract

The production of nonindustrial cannabis is highly developed in the Moroccan Rif region; however, local farmers consider hemp seeds which are rich in omega 3 and tocopherols, only as by-products of cannabis cultivation with low market value. The local ecotype is considered to be a plant with a cannabinoid content of more than 0.4%. So, the objective of this research is to investigate how the incorporation of this local hemp seed affects productive performance and egg quality traits. The experiment is conducted to evaluate the effects of hemp seed (HS) incorporation on hen laying performance and physical egg quality at three levels: 10% (HS-10% group), 20% (HS-20% group), and 30% (HS-30% group). Ninety-six Lohmann Brown classic laying hens were randomly assigned to a control group and three feed treatments. The sampling was taken after the 28-week rearing period (peak egg laying). Throughout the experiment, low-rate HS inclusion (HS-10%) showed no significant differences in egg-laying performance (*p* > 0.05). However, the high incorporation rates of HS (20% and 30%) negatively affected the egg-laying performance (84–94% and 80–86%, respectively). The albumen quality was also improved by the HS inclusion, where the highest values of the Haugh unit were recorded, ranging between 68.69 and 73.91 for the HS-30% groups. The results also show that HS inclusion and duration influence significantly the yolk color (*p* < 0.001). The yellow intensity decreases with HS incorporation and aging, from a dark yellow (*b*^*∗*^ = 38.63 for the control group) to a very pale yellow (*b*^*∗*^ = 26.29 for HS 30% group). Based on these findings, we can conclude that the incorporation of nonindustrial Moroccan cannabis seeds (ecotype *Beldiya*) at low rate in the diet of laying hens does not alter the laying performance or the quality of the egg; therefore, they could be used in poultry feeding as an alternative constituent to partially replace high-cost imported ingredients, such as corn and soybeans.

## 1. Introduction

Egg production is one of the most important poultry industries, attracting substantial investment worldwide. Consequently, there is an urgent need to improve layer productivity and egg quality by using natural feed substances [1, 2]. Consumers have become more aware and demand healthy foods that are exempt from synthetic substances [3]. Using phytogenic extracts, as a source of bioactive compounds, is becoming an appealing strategy to improve the health-related properties of animal-source foods (ASF) [4]. To accomplish this requirement, food scientists and animal breeders are developing different strategies to improve the nutritional profile of ASF. The latter are important sources of essential amino acids, vitamins, and oligoelements, but their lipid composition is often criticized. Enriching animal products with n-3 PUFAs remains a sustainable solution for a healthy diet [5]. Furthermore, the use of phytogenic feed additives is becoming an attractive and sustainable strategy to improve animal products' lipid quality and functional properties.

Drought has always been present in the history of Morocco, but its importance as a structural element of the country's climate has been increasing in recent decades. The cultivation of oilseeds is often very dependent on irrigation; consequently, the production of these seeds is very limited. Morocco is indeed 80% dependent on imports. It has a significant deficit in oilseeds, which are an essential ingredient in poultry feed. Oilseed crops partially contribute to the country's food security by producing edible oils and providing cakes which are protein-rich by-products and essential for the animal feed industry (e.g., the poultry sector). In the current context of drought, war, and the Covid pandemic, the price of oilseeds has soared, leading to an increase in the price of poultry feed and, therefore, chicken and eggs.

Cannabis (*Cannabis sativa* L.) is one of the oldest and most versatile herbal plants cultivated by man. This plant has traditional and cultural roots in many countries around the world. Recent publications suggest that it originated in Central Asia [6, 7], while others suggest its origins are in South Asia [8]. Cannabis seeds that have been unused in recent years by cannabis producers in Morocco could constitute an alternative source, even partial, to soybeans, sunflower, and maize seeds imported at increasingly high prices. The hemp seed and its cake represent an interesting source of amino acids, phenols, tocopherols, minerals, and polyunsaturated fatty acids [9]. It was demonstrated in our previous study that the local hemp seed contains 94.08% of dry matter, including 20–23% crude protein, 30–34% lipids (80% of which are polyunsaturated fatty acids), 26–37% insoluble fiber, and 3–5% ash [10]. These characteristics imply its potential use as a phytogenic ingredient or an additive in animal feed with health-promoting properties. So, hemp seeds could be a valuable alternative to improve and reduce the cost of imported feeding stuff for animals, mainly poultry. In fact, nonindustrial hemp seeds, considered a by-product of cannabis culture, have a low commercial value. However, they have excellent nutritional value in terms of lipid profiles. According to the United Nations Office on Drugs and Crime, there were 47.196 hectares of hemp cultivation in Moroccan Rif [11]. It was prohibited because of the psychoactive and addictive properties of its tetrahydrocannabinol (Δ9-THC) component. However, in December 2020, in order to take advantage of the growing legal market and increase farmers' income, the Moroccan government initiated the legalization and regulation of cannabis production with low THC for innovative industrial applications. Actually, there is a growing interest in the plantation of cannabis for medicinal and cosmetic uses, and even in the use of whole hemp seeds in food as well as in the production of flour and hemp seed oil. Much research has been performed on adding hemp to animal feed. Due to its positive effects, much attention has been oriented recently to the use of Cannabis sativa in farm animal diets, especially poultry [12]. However, the effect of the Moroccan ecotype of Cannabis sativa seeds was never studied.

The ultimate goal of the research is to promote the Moroccan hemp seeds produced in the Rif by including them in poultry diets. With this in mind, this study aims to evaluate the effect of diets based on different incorporation rates of these seeds on egg production and quality traits.

## 2. Material and Methods

### 2.1. Birds and Housing

The animal experiment was conducted at the Royal Institute of livestock, Kenitra, Morocco, in compliance with the European Code CO 74/99 concerning stocking density, lighting, vaccination, and other procedures. No breeding practices other than those normally employed were introduced during the trial.

Ninety-six (*n* = 96) Lohmann Brown Classic (LBC) laying hens were randomly assigned to three feed treatments and a control group; each treatment was repeated 6 times (4 Hens/replicates). The hens used in this experiment were selected from a legitimate industrial farm at 22 weeks of age with an initial weight varying between 1.70 and 1.80 kg. The birds were carried out in a semi-automatic facility with automatic dung mats; whereas, the hens were housed, 4 per cage, equipped with trough feeders and nipple drinkers with the following dimensions: 61 cm long, 57 cm wide, and 50 cm high. The hens were confined in optimal conditions with temperature and humidity controlled at 18–20°C and 55–60%, respectively. The lighting regimen was 16 h/day from 06:00 am to 10:00 pm. The laying hens were kept in welfare conditions.

### 2.2. Diets and Experimental Approach

Diets were formulated using similar nutrients and ingredients to standardize the energy level of the studied diets (3000 kcal/kg). The formulation was developed in consultation with BENWAY, a company specializing in poultry feed. Maize/soya bean-based diets were utilized by the inclusion of different levels of hemp seed of the local ecotype “Beldiya: 0.0% (HS-0), 10% (HS-10), 20% (HS-20), and 30% (HS-30). At 22 weeks of age, all hens were allowed to adapt to the new environment system (cage and feed) for 2 weeks. During the first week, hens were fed a commercial layer diet, and during the second week, the studied diets were progressively incorporated (25%, 50%, 75%, then 100%). Feed was distributed 3 times per day, and the water was made *ad libitum* to the hens. Eighteen eggs per group were sampled randomly during the last three days of each period 24–28, 28–32, and 32–36 for egg quality traits and yolk color measurement. The chemical compositions of the HS and experimental diets used in this study are given in Tables 1 and 2, respectively.

### 2.3. Hen Performance

In total, 216 eggs were collected for the 4 study groups, which corresponds to 18 eggs per experimental group for 3 days, resulting in 54 eggs for each treatment. Egg production (EP) and egg weight (EW) were measured and recorded daily. Total feed consumption was determined as the difference between feed offered and residual feed remaining in the feeders (g feed offered/g feed remaining). Hen body weight (BW) was measured weekly, on the same day as the weight of the feed. Feed conversion ratio (FCR) per dozen eggs was calculated by recording the total feed consumed in a week divided by the total number of eggs produced in that week and multiplied by 10 [13]. Mortality was recorded daily.

### 2.4. Egg Quality Measurements

For external and internal egg quality parameters, nine eggs were hand collected from each cage and enumerated. Each egg was weighed using an electronic balance *(Radwag Wagi Elektroniczne).* Eggs were broken on a flat glass surface, and eggshell weight (g) was measured. The albumen and yolk were separated, the yolk was weighed, and the thick albumen and egg yolk height was measured within an electronic tripod micrometer (*Ingco-HDCD01200*). The albumen and yolk weights were divided by the whole egg weight and then multiplied by 100 to determine the weight percentage. Egg shape index (%), egg yolk index (%), and egg yolk albumen index (%) were recorded according to Romanoff and Romanoff [14]. Haugh units were calculated using the following formula [15]:

(1) $$\begin{array}{c} \left[HU=100\;\log\;\left(H-1.7W^{0.37}+7.57\right)\right]\text{,} \end{array}$$

where *H* represents the white height and *W* represents the egg weight.

### 2.5. Color Yolk Analysis

The yolk color was measured using Chroma meter (Konica Minolta, CR400) according to the CIELAB system: *L*^*∗*^ lightness, *a*^*∗*^ redness (red-green), and *b*^*∗*^ yellowness (blue-yellow). The hue angle *H*^*∗*^ and chromaticity *C*^*∗*^ were calculated by the following formula [16]:

(2) $$\begin{array}{c} H^{*}=\left({\left(\arctan\frac{b*}{a*}\right)}^{*}57.29\right)+180;\;C^{*}=\sqrt{\left(a^{2}+b^{2}\right)}\text{.} \end{array}$$

### 2.6. Statistical Analysis

Statistical Package for the Social Sciences (IBM SPSS. 21) was used to perform the statistical analyses. The normal distribution was checked according to the Shapiro–Wilk test. A two-way analysis of variance (ANOVA) was performed for zootechnical performance, physical quality of eggs, and yolk color. Tukey's post hoc test was used for comparison of means. The difference was considered significant at *p* < 0.05. A principal component analysis (PCA) was performed on the data set to differentiate the results according to the age of the hens and the feed distributed.

## 3. Results and Discussion

### 3.1. Zootechnical Performances

The effect of hemp seed incorporation on production efficiency and egg quality has been the subject of several works, some of which revealed a beneficial effect while others showed the opposite [17]. However, most research focused on industrial cannabis, whereas our experiment examined the effects of seeds devoid of THC that originated from ecotype *Beldiya*, which is categorized as a nonindustrial variety. The effects of the Moroccan ecotype Beldiya incorporation on the zootechnical performance, given in Tables 3–6, show that the hemp seed diet and duration considerably impact all the examined zootechnical parameters (*p* < 0.05). The degree of the seeds' inclusion has a positive effect at specific levels which should not be exceeded. The highest levels of egg production (100%) were observed in the HS-10 treatment during the first week, while the lowest levels were observed in the HS-30 treatment during the same week (80.56%). This study was carried out at the peak of egg laying (28–33 weeks of age) of the LBC strain according to the standard of this strain [18]. However, the pre-registered laying peaks in our experimentation were different from those of the treatments, indicating the effect of the incorporation rate of HS on hen laying peaks. The peak of oviposition was recorded early in the W-28 for the HS-10% group with 100% of egg production, and then it stabilizes at 97.22%. On the other hand, the control group as well as the HS-20% group reached their intermediate peaks in the W-32 with 100% and 94.44%, respectively. In contrast, the peak egg production was recorded late for HS-30% in W-36 with 86.11%, below the peak of the Lohmann Brown Classic strain determined at 94%. This result could be explained by the different adaptation periods required for each studied group. Skřivan et al. [19] showed that the use of low doses of hemp seed in poultry feed can improve performance and product quality, but more than 30 g/kg can lead to poor performance due to antinutritional agents, including polyphenols and phytate. Indeed, if greater amounts of cannabis seeds are added, the adaption period is expected to be prolonged. The recorded effects of the HS diet are comparable to those reported in Neijat et al. [20], where a study was conducted on the effect of industrial HS on the zootechnical traits of laying hens. The findings demonstrate that regardless of the dosage of cannabis seeds used, EW rises with age. Thus, the EW increases on average from 55 to 60 g (control), 53 to 56 g (HS 10%), 50 to 59 g (HS 20%), and 52 to 58 g (HS 30%). However, adding cannabis seeds to the feed did not result in an increase in egg weight compared to the control. Thus, after 32 weeks, the weight of the eggs decreases from control to 30% HS by an average of 10 g. These results are in accordance with [20], whose findings demonstrated that hens receiving 30% HS had significantly lower EW than the control or lower HS doses (10 or 20%). [21] worked on 19-week-old hens with two weeks of adaptation, showing that egg weights increased between the first and second weeks; the study also reported a steady EW increase until the fifth week before eventually remaining constant. However, March and MacMillan [22] demonstrated that a linolenic acid increase in the diet was an important factor in improving egg size and weight. This finding can be explained by the need for long-chain fatty acids to synthesize lipoproteins, which can be transported to the ovary to be absorbed by developing eggs (March and MacMILLAN, 1990). The observed increase in egg production and egg mass of laying hens in the present investigation for HS-10% was probably due to the presence of essential nutrients in hemp seeds such as amino acids, long-chain fatty acids, oligo, and microelement components, and fibers in this phytogenic resource that has resulted in better laying performance at a low rate. It is also possible that hemp seeds might have stimulated the hepatic secretion of egg yolk precursors by protecting hepatocytes from oxidative damage with subsequent enhancement of yolk formation and ovulation [4]. In this sense, Gharaghani et al. [23] demonstrated that using fennel (*Foeniculum vulgare*) in poultry feeding, particularly in laying hens, decease the heat stress. Based on this proposition, this coproduct of cannabis culture can replace the chemical hepatoprotector used in laying hens. These results are in agreement with those reported by Lee et al. [24], which showed that hempseeds meal rich with PUFA can reduce oxidative toxicity due to their antioxidant properties.

The hens' weight declines after 32 weeks but grows during the 36^th^ week. Depending on experiment duration, these variations would be most explained partly by the more or less rapid adaptation to diet-supplemented cannabis seeds and by the laying rate. In the control group, the variation in hen's weight could be explained by the increase in the laying rate, which accounts for a large part of the energy consumed. The weight increased after the decline in the laying rate. Jing et al. [21] and Silversides and Lefrancois [25] observed a reduction in hen body weight over their study period regardless of the inclusion levels. Regarding the FI and FC, the results also show a significant difference (*p* < 0.05) between the studied groups. The hen's feed intake showed a marked increase between W-28 and W-32 and a stability between W-32 and W-36 for all studied groups except for the HS-30% group. This increase positively affects the FC, logically leading to an increase in egg weight throughout the experiment. These results could be linked to the adaptation of the hens to the feed ration and to their increase in body weight. Also, they can be related to the increase in egg weight. We also noted that FI was greater than 140 g/egg/day regardless of the duration of the experiment or the seed dose included in the diet, indicating that the addition of cannabis seeds did not affect the diet palatability.

### 3.2. Physical Egg Quality

The results of the hemp seed inclusion in the laying diet are illustrated in Tables 4 and 6. The YW was negatively correlated with the incorporation rate of HS (*p* < 0.05), but positively correlated with the aging period (Table 4). The higher value of YW was recorded in the egg of the control group at W28 (25.61%), while the lowest value was found for HS-30 at W28 (21.38%). The AW was not affected by the duration for the control and HS-10% groups, but a decrease was recorded between W-32 and W-36 for HS-20% and between W-28, W-32, and W-36 for HS-30%. However, the lowest results were recorded for the control and HS-10% group at W-28 and W-36 (64.28% and 64.38%, respectively). The highest result was registered at W-28 and W-32 for HS-30% and HS-20% groups. Cufadar et al. [26] reported that adding hemp seed meal to the diet of quails increases the albumen index. This result could be linked to the same bioactive components of HS, such as antioxidants, crude protein, and long-chain fatty acids that protect the magnum and uterus and promote albumin secretion in layers [27]. Haugh unit (HU) was calculated using the albumen height and egg weight. It is a parameter that makes it possible to evaluate the albumen quality [4]. The current study results show that the inclusion of HS in a hen's diet significantly improves albumen quality (HU). The HU in the HS-30% group was significantly higher than other groups, as depicted by the following results: 73.91 for HS-30, 73.91 for HS-10, 72.54 for HS-20, and 68.03 for control; all results are registered at W36. Furthermore, the HU was affected by age (*p* < 0.05). This improvement could be related to the HS richness of the high-quality protein, especially the edestin and albumin fractions. This result is in agreement with that reported by [28], which explains this improvement of HU by the HS inclusion rate. Moreover, several studies demonstrate that using phytogenic additives with antibacterial and antioxidant properties in laying hen diets could improve albumen quality [29, 30]. The Yolk index (YI) showed no difference between HS-10 and control (*p* > 0.05). The average of the recorded index for the other groups was around 0.43. The best results were recorded at W32 for HS-20 and HS-30 by an overall average of 0.48 and 0.47, respectively. Our data show that all the eggs tested are in the extra-fresh class (YI > 0.38). In addition, the incorporation rate of HS is positively correlated with the YI increasing and will consequently provide an improvement in egg quality and contribute to improving the conceivability of eggs. Our results are similar to those of Hosseini-Vashan and Ghiasi [31] who reported that an increase in the inclusion of industrial hemp seeds or hemp seed oil leads to a significant improvement in the yolk index.

Concerning the shell thickness (ST), HS 10% is similar to control for all age groups. HS 20% and HS 30% have a higher value than control for only 36 weeks of age with an average of 0.44 mm. This result could be explained by the adaptation phenomenon and the effect of the accumulation of bioactive molecules.

### 3.3. Yolk Color

Although there are differences of opinion around the world regarding consumer preference for egg yolk color, all studies agree that this parameter is among the most important criteria in the purchase of eggs [32]. As shown in Table 5, the yolk color varies according to the duration of the experiment and the dose of cannabis seeds included in the diet. Thus, the comparison of eggs from control hens and those from hens on diets with cannabis seed supplementation (10, 20, and 30%) shows significant differences (Tables 5 and 6) in the color parameters: *L*^*∗*^ (lightness), *a*^*∗*^ (redness), and *b*^*∗*^ (yellowness). The most visible effect is the decrease of *b*^*∗*^, which considerably affects the intensity of the yellow color, changing from a dark yellow to a very pale yellow. Indeed, after 36 weeks of experimentation, *b*^*∗*^ goes from a value of 38.63 (control) to 26.29 (H-S 30%). This decrease can be explained by the fact that the control lot hens were fed a diet containing corn seeds rich in carotenoids, and that they were gradually replaced by cannabis seeds whose carotenoid content was much lower than that of corn. Maoka [33] linked the dark yellow color to the content of carotenes and xanthophylls, while the orange-red color would be linked to chlorophylls. This decrease in yolk intensity may be a handicap for marketing these eggs on the Moroccan market, as consumers prefer eggs with an intense yellow yolk color, which they generally link to a free-range diet. It will be necessary to think about improving the quality of the eggs produced by including doses of cannabis not exceeding 10% or by another formulation where the cannabis would be a substitution of soya or sunflower cakes and not of corn.

### 3.4. Principal Component Analysis

**Figure 1:**
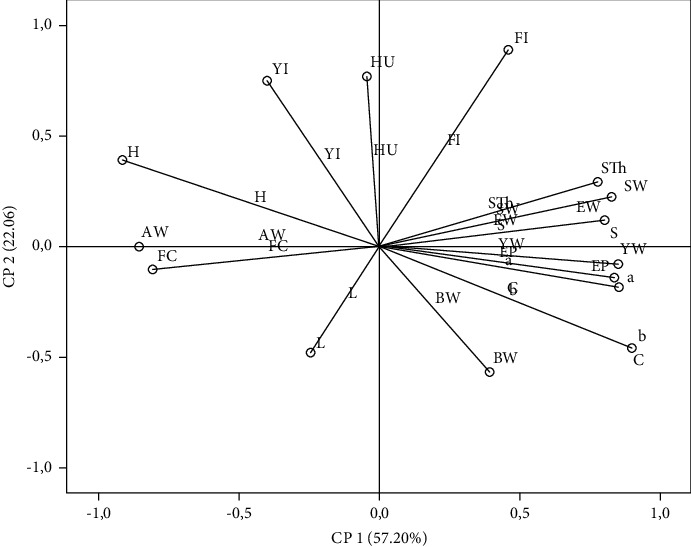
Projection of zootechnical performance, physical egg quality, and yolk color measurement in the plane defined by two principal components. YW: yolk weight; AW: albumen weight; *a*^*∗*^: redness; *H*^*∗*^: hue angle; *b*^*∗*^: yellowness; *C*^*∗*^: chromaticity; EP: egg production; STh: shell thickness; EW: egg weight; S: surface; FC: feed consumption; SW: shell weight; HU: haugh unit; YI: yolk index; FI: feed index; and BW: body weight.

Principal component analysis (PCA) was carried out to easily visualize the relationships among the studied groups of hens and evaluated parameters. The analysis was conducted using 18 variables, including zootechnical performance, egg quality traits, and yolk color measurement. The results show that the three first principal components (PC) account for more than 85% of the total information (Table 7). These three PC explained 57.20%, 22.06%, and 6.63%, respectively. The PC1 was mainly characterized by shell weight, egg weight, egg percentage, and yellowness index on the right side. The left side was characterized by albumen weight and feed conversion (FC), Figure 1. The PC2 was defined positively by feed intake (FI), Haugh units (HU), and the yolk index, and in the opposite direction, it was defined by lightness and body weight (BW).

**Figure 2:**
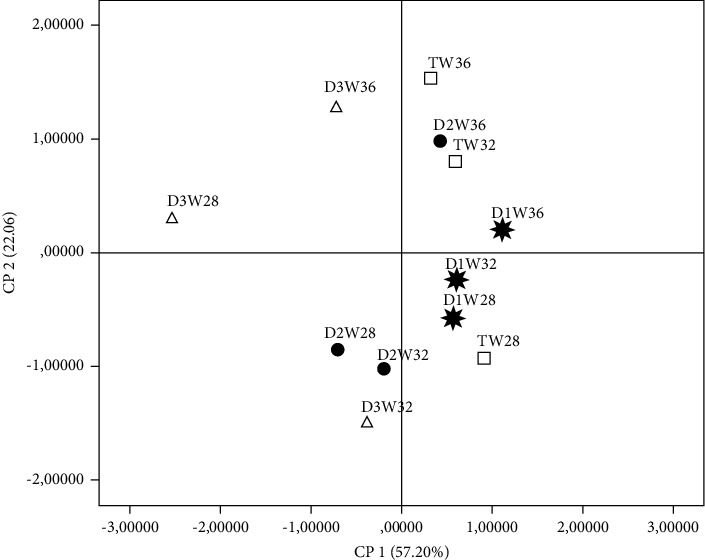
Projection of the variables of the four studied groups in the plane defined by two principal components. TW28: control week 28; TW32: control week 32; TW36: control week 36; D1W28: HS-10% week 28; D1W32: HS10% week 32; D1W36: HS-10% week 36; D2W28: HS-20% week 28; D2W32: HS-20% week 32; D2W36: HS-20% week 36; D3W28: HS-30% week 28; D3W32: HS-30% week 32; and D3W36: HS-30% week 36.

The projection of groups on the factorial map reveals a discrimination between the studied hen groups and the measured parameters (Figure 2). This discrimination allows a simple and summarized interpretation already mentioned above by ANOVA analysis. In fact, the control and the HS-10% were located on the right side of Figure 2 and were clearly differentiated from all studied hens, where the EP, EW, and yellowness index lie. The HS-30% groups were differentiated from the other hen types and are located on the left side of Figure 2. Concerning PC 2, the relationship between the studied hen groups is unclear. The two most important variables, which are regrouped into the PC 2, were HU and FI. The PCA exposed the effect of Moroccan autochthonous nonindustrial hemp seed incorporation in the diet of laying hens on zootechnical performance and physical egg quality. Numerous researchers reported similar discrimination between animals reared under phytogenic-based feeding [34, 35].

## 4. Conclusion

This study investigates the effect of the Moroccan ecotype nonindustrial hemp seed incorporation in the laying hens' diet on zootechnical performance and physical egg quality. The results show that the incorporation of hemp seed at low doses (<20%) has no adverse effect on the laying performance. Furthermore, incorporating HS in the diet leads to improved albumen quality while the yolk index was not affected. However, this incorporation causes a diminution of the egg yolk's color intensity. Based on these conclusions, Moroccan ecotype nonindustrial hemp seeds can be introduced at low rates as a substitute for conventional ingredients of laying hen feed, such as corn, soybeans, or other oil seeds. Further studies are being conducted in our laboratory to evaluate the nutritional value of the eggs produced as a result of this incorporation.

## Figures and Tables

**Table 1:** Chemical composition of hemp seed ecotype *Beldiya* used in this study.

Constituents	Fresh matter
Dry matter (%)	88.27
Total phosphorus (g/kg)	11.60
Crude protein (%)	22.00
Total lipids (%)	33.00
Calcium (g/kg)	1.725
Humidity (%)	11.73
Mineral matter (g/kg)	49.70
Crude cellulose (%)	14.68

**Table 2:** Diet composition and its calculated nutritional value.

	Control	HS-10%	HS-20%	HS-30%
*Diet ingredient*				
Hemp seed	0	10	20	30
Sunflower meal	1.685	13.000	13.000	13.000
Soybean meal	25.585	10.459	9.563	11.094
Calcium	8.609	7.379	8.934	8.927
DDGS	0.896	7.000	7.000	3.572
Corn	58.829	46.214	38.001	21.294
Dicalcium phosphate	1.368	3.726	1.162	2.000
Soybean oil	2.028	1.000	0.000	0.000
Premix 1	0.500	0.500	0.611	8.000
Sodium sulfate	0.000	0.237	0.300	0.200
Salt	0.183	0.112	0.200	0.200
DL-methionine	0.273	0.215	0.217	0.200
L-lysine HCl	0.004	0.146	0.999	1.500
Premix 2	0.040	0.012	0.013	0.013
Total	100	100	100	100
Dry matter (%)	88.312	88.536	86.682	81.242

*Calculated nutritional composition*				
Metabolizable energy (kcal kg^−1^)	2989.920	3000.000	3000.002	3000.003
Humidity (%)	11.195	10.972	10.352	9.537
Crude protein (%)	17.799	17.800	18.000	18.000
Total lipids (%)	5.057	7.143	8.480	11.102
Ash (g/kg)	126.960	140.863	189.627	244.972
Calcium (g/kg)	38.996	40.000	40.000	40.000
Phosphorus available (g/kg)	4.396	8.510	27.762	27.752
Sodium (g/kg)	0.083	0.160	0.160	0.160
Linoleic acid (%)	2.491	7.454	12.092	17.370
Lysine (g/kg)	9.352	8.642	8.705	8.735
Methionine (g/kg)	5.580	5.589	5.823	6.138
Leucine (g/kg)	14.921	14.528	14.140	13.522
Methionine + cysteine (g/kg)	8.779	8.638	8.576	8.616
Threonine (g/kg)	6.902	6.634	6.944	6.913
Tryptophan (g/kg)	1.760	1.826	1.944	1.917

DDGS, distillers dried grains with soluble; premix 1, vitamin premix; premix 2, mineral premix.

**Table 3:** Effect of nonindustrial hemp seed incorporation in the diet of laying hens on zootechnical performance during the experimental period.

Parameters	Treatments											
	Control			HS-10%			HS-20%			HS-30%		
	W-28	W-32	W-36	W-28	W-32	W-36	W-28	W-32	W-36	W-28	W-32	W-36
Egg production (%)	93.05^bcd^ ± 8.19	100^d^ ± 7.45	98.61^d^ ± 3.40	100^d^ ± 0.00	97.22^d^ ± 4.30	97.22^cd^ ± 4.30	84.72^a^±3.40	94.44^c^ ± 8.60	87.50^b^ ± 4.56	80.56^a^ ± 7.45	81.94^a^ ± 6.27	86.11^ab^ ± 6.80
Egg weight (g)	55.05^cd^ ± 3.32	59.47^ef^ ± 2.59	60.06^f^ ± 1.55	53.89^c^ ± 2.06	55.26^cd^ ± 1.73	56.94^de^ ± 1.61	50.99^ab^ ± 1.35	52.50^abc^ ± 2.00	59.25^ef^ ± 1.84	52.52^bc^ ± 2.88	49.86^a^ ± 2.82	58.21^ef^ ± 3.36
Feed intake (g/hen/day)	106.9^a^ ± 1.62	119^c^ ± 0.13	118.5^c^ ± 0.44	108.36^b^ ± 1.01	118.95^c^ ± 0.27	118.41^c^ ± 0.4	107.48^ab^ ± 1.12	118.2^c^ ± 0.78	117.81^c^ ± 0.81	108.8^b^ ± 3.10	118.19^c^ ± 0.67	117.91^c^ ± 0.39
Feed conversion	1.82^cde^ ± 0.10	1.68^a^ ± 0.10	1.67^a^ ± 0.04	1.86^de^ ± 0.06	1.81^bcd^ ± 0.05	1.76^abcd^ ± 0.04	1.97^fg^ ± 0.05	1.91^ef^ ± 0.07	1.69^a^ ± 0.05	1.92^ef^ ± 0.10	2.02^g^ ± 0.11	1.73^ab^ ± 0.06
Body weight (kg)	1.84^e^ ± 0.04	1.76^f^ ± 0.03	1.83^e^ ± 0.03	1.75^f^ ± 0.04	1.67^c^ ± 0.05	1.7^c^ ± 0.04	1.66^bc^ ± 0.04	1.57^a^0.02	1.61^ab^ ± 0.05	1.69^c^ ± 0.04	1.61^ab^ ± 0.05	1.68^c^ ± 0.05
Mortality	00	00	00	00	00	00	00	00	00	00	00	00

W-28: Week 28; W-32: Week 32; W-36: Week 36; HS-10%: hemp seed 10%; HS-20%: hemp seed 20%; HS-30%: hemp seed 30%. Mean ± standard deviation. Significant differences (*p* < 0.05) between means are shown with different lowercase letters (a–f).

**Table 4:** Effect of nonindustrial hemp seed incorporation in the diet of laying hens on physical egg quality during the experimental period.

Parameters	Treatments											
	Control			HS-10%			HS-20%			HS-30%		
	W-28	W-32	W-36	W-28	W-32	W-36	W-28	W-32	W-36	W-28	W-32	W-36
Yolk weight (%)	25.61^e^ ± 1.41	24.85^cde^ ± 0.83	24.93^cde^ ± 1.27	23.95^bcd^ ± 0.9	24.64^bcde^ ± 1.30	25.26^de^ ± 1.33	23.55^bc^ ± 0.68	23.9^bcd^ ± 0.62	25.00^cde^ ± 0.88	21.38^a^±1.74	23.28^b^ ± 1.38	24.08^bcde^ ± 1.40
Albumen weight (%)	64.28^a^ ± 1.17	64.7^ab^ ± 0.83	64.63^ab^ ± 1.27	65.91^abc^ ± 0.93	65.07^abc^ ± 1.23	64.38^a^ ± 1.38	66.61^c^ ± 0.75	66.65^c^ ± 1.46	64.87^ab^ ± 0.90	69.00^d^ ± 1.98	66.22^bc^ ± 1.78	65.41^abc^ ± 1.47
Haugh units	61.45^a^ ± 3.69	63.69^ab^ ± 7.28	68.03^bcd^ ± 4.92	66.41^abc^ ± 5.64	68.73^bcd^ ± 5.33	73.41^d^ ± 2.81	61.67^a^ ± 6.02	71.23^cd^ ± 3.82	72.54^cd^ ± 6.81	68.69^bcd^ ± 4.15	73.64^d^ ± 3.81	73.91^d^ ± 3.29
Yolk index	0.42^a^ ± 0.01	0.43^abcd^ ± 0.01	0.44^abcd^ ± 0.01	0.43^a^ ± 0.01	0.42^ab^ ± 0.01	0.42^abc^ ± 0.01	0.43^ab^ ± 0.01	0.48^f^ ± 0.02	0.45^cd^ ± 0.01	0.44^bcd^ ± 0.01	0.47^ef^ ± 0.10	0.46^de^ ± 0.01
Shell thickness, mm	0.39^bcd^ ± 0.02	0.41^e^ ± 0.01	0.44^f^ ± 0.01	0.41^de^ ± 0.01	0.40^bcde^ ± 0.00	0.43^f^ ± 0.01	0.38^bc^ ± 0.02	0.38^b^ ± 0.01	0.43^f^ ± 0.01	0.34^a^ ± 0.01	0.40^cde^ ± 0.01	0.43^f^ ± 0.01

W-28: Week 28; W-32: Week 32; W-36: Week 36; HS-10%: hemp seed 10%; HS-20%: hemp seed 20%; HS-30%: hemp seed 30%. %. Mean ± standard deviation. Significant differences (*p* < 0.05) between means are shown with different lowercase letters (a–f).

**Table 5:** Effect of nonindustrial hemp seed incorporation in the diet of laying hens on yolk color measurement during the experimental period.

Parameters	Treatments											
	Control			HS-10			HS-20			HS-30		
	W-28	W-32	W-36	W-28	W-32	W-36	W-28	W-32	W-36	W-28	W-32	W-36
Lightness (*L*^*∗*^)	57.09^c^ ± 1.07	54.99^ab^ ± 1.10	53.35^a^ ± 1.30	56.15^b^ ± 0.91	55.34^ab^ ± 0.68	53.67^ab^ ± 1.82	54.18^ab^ ± 1.03	54.28^ab^ ± 1.19	53.27^a^ ± 2.13	54.38^ab^ ± 1.21	55.29^b^ ± 1.05	53.88^ab^ ± 1.54	
Redness (*a*^*∗*^)	−4.48^cd^ ± 0.99	−4.32 ^cd^ ± 0.66	−4.13^d^ ± 0.25	−4.36^cd^ ± 0.63	−4.84^c^ ± 0.27	−3.87^d^ ± 0.54	−6.19^ab^ ± 0.14	−5.97^ab^ ± 0.16	−3.89^d^ ± 0.65	−6.38^a^ ± 0.09	−5.77^b^ ± 0.09	−6.33^ab^ ± 0.10	
Yellowness (*b*^*∗*^)	37.89^e^ ± 4.36	39.59^ef^ ± 2.72	38.63^ef^ ± 2.27	41.70^f^ ± 3.43	34.35^d^ ± 2.15	40.06^ef^ ± 2.13	28.77^c^ ± 1.68	19.98^b^ ± 1.75	37.75^e^ ± 2.98	15.12^a^ ± 0.86	13.26^a^ ± 0.94	26.29^c^ ± 2.77	
Hue angle (*H*^*∗*^)	97.07^ab^ ± 2.16	96.38^a^ ± 1.50	96.25^a^ ± 0.64	96.09^a^ ± 1.29	98.11^b^ ± 0.81	95.59^b^ ± 1.01	102.39^c^ ± 0.83	107.30^e^ ± 1.41	96.01^a^ ± 1.03	112.97^f^ ± 0.94	113.75^e^ ± 1.08	104.00^d^ ± 1.86	
Chromaticity (*C*^*∗*^)	38.20^e^ ± 4.23	39.84^ef^ ± 2.62	38.87^ef^ ± 2.24	41.94^f^ ± 3.36	34.70^c^ ± 2.12	40.26^ef^ ± 2.08	29.45^c^ ± 1.64	20.89^b^ ± 1.69	37.96^e^ ± 2.96	16.42^a^ ± 0.83	14.47^a^ ± 0.90	27.06^c^ ± 2.66	

W-28: Week 28; W-32: Week 32; W-36: Week 36; HS-10%: hemp seed 10%; HS-20%: hemp seed 20%; HS-30%: hemp seed 30%. %. Mean ± standard deviation. Significant differences (*p* < 0.05) between means are shown with different lowercase letters (a–f).

**Table 6:** Analysis of variance for the effect of nonindustrial hemp seed incorporation in the diet of laying hens on zootechnical performance, physical egg quality, and yolk color measurement during the experimental period.

	d*f*	Mean squares	*F*-value	*p* value
*Egg production (%)*				
Dose	3	936.21	27.08	<0.001
Period	3	158.07	4.57	<0.01
Dose × period	6	68.09	1.97	>0.05
Error	59	34.56		

*Egg weight (%)*				
Dose	3	74.15	14.63	<0.001
Period	3	134.78	26.69	<0.001
Dose × period	6	27.23	5.93	<0.001
Error	59	5.05		

*Feed intake (g/hen)*				
Dose	3	31.74	1.45	>0.05
Period	3	8978.80	411.46	<0.001
Dose × period	6	48.06	2.20	>0.05
Error	59	21.82		

*Feed conversion*				
Dose	3	0.08	14.85	<0.001
Period	3	0.14	26.72	<0.001
Dose × period	6	0.03	5.63	<0.001
Error	59	0.006		

*Yolk weight (%)*				
Dose	3	16.91	49.20	<0.001
Period	3	18.20	59.95	<0.001
Dose × period	6	2.18	6.36	<0.001
Error	59	0.34		

*Albumen weight (%)*				
Dose	3	14.21	3.99	<0.05
Period	3	37.83	10.64	<0.001
Dose × period	6	12.27	3.45	<0.01
Error	59	3.55		

*Haugh unit*				
Dose	3	179.75	7.16	<0.001
Period	3	256.05	10.20	<0.001
Dose × period	6	22.31	0.89	>0.05
Error	59	25.08		

*Yolk index*				
Dose	3	0.005	15.97	<0.001
Period	3	18.200	52.95	<0.001
Dose × period	6	0.001	3.15	<0.05
Error	59	0.000		

*Shell thickness, mm*				
Dose	3	0.002	5.93	<0.01
Period	3	0.010	39.23	<0.001
Dose × period	6	0.002	4.76	0.001
Error	59	0.000		

*Lightness (L* ^*∗*^)				
Dose	3	4.63	2.67	>0.05
Period	3	11.57	6.68	<0.01
Dose × period	6	3.81	2.20	>0.05
Error	59	1.73		

*Redness (a* ^*∗*^)				
Dose	3	13.63	57.50	<0.001
Period	3	2.98	12.57	<0.001
Dose × period	6	2.51	10.62	<0.001
Error	59			

*Yellowness (b* ^*∗*^)				
Dose	3	1656.45	254.92	<0.001
Period	3	445.12	68.50	<0.001
Dose × period	6	128.58	19.78	<0.001
Error	59	6.49		

*Hue angle (H* ^*∗*^)				
Dose	3	718.61	426.76	<0.001
Period	3	181.51	107.79	<0.001
Dose × period	6	52.06	30.92	<0.001
Error	59	1.68		

*Chromaticity (C* ^*∗*^)				
Dose	3	1526.46	247.17	<0.001
Period	3	415.85	67.33	<0.001
Dose × period	6	119.62	19.37	<0.001
Error	59	6.17		

**Table 7:** Three main components explain more than 85% of the total information on zootechnical performance, physical egg quality, and yolk color measurement during the experimental period.

Variables	Principal component			Variables	Principal component		
	1	2	3		1	2	3
YW	0.925	0.219	0.022	EW	0.432	0.885	−0.026
AW	−0.907	−0.288	−0.020	S	0.433	0.884	−0.028
*a* ^*∗*^	0.831	0.304	−0.154	FC	−0.446	−0.876	0.039
*H* ^*∗*^	−0.813	−0.391	0.344	SW	0.526	0.807	0.072
*b* ^*∗*^	0.792	0.381	−0.416	HU	−0.114	0.208	0.863
*C* ^*∗*^	0.789	0.382	−0.421	YI	−0.363	−0.084	0.807
EP	0.770	0.181	−0.201	FI	0.293	0.400	0.779
STh	0.622	0.594	0.337	BW	0.316	0.288	−0.752

YW: yolk weight; AW: albumen weight; *a*^*∗*^: redness; *H*^*∗*^: hue angle; *b*^*∗*^: yellowness; *C*^*∗*^: chromaticity; EP: egg production; STh: *shell* thickness; EW: egg weight; S: surface; FC: feed consumption; SW: shell weight; HU: haugh unit; YI: yolk index; FI: feed index; BW: body weight.

## Data Availability

The original data from the paper are available from the corresponding author upon reasonable request.
